# Bovine Leptospirosis: Serology, Isolation, and Risk Factors in Dairy Farms of La Laguna, Mexico

**DOI:** 10.3390/life15081224

**Published:** 2025-08-02

**Authors:** Alejandra María Pescador-Gutiérrez, Jesús Francisco Chávez-Sánchez, Lucio Galaviz-Silva, Juan José Zarate-Ramos, José Pablo Villarreal-Villarreal, Sergio Eduardo Bernal-García, Uziel Castillo-Velázquez, Rubén Cervantes-Vega, Ramiro Avalos-Ramirez

**Affiliations:** 1 Cuerpo Académico de Epidemiología Veterinaria, Facultad de Medicina Veterinaria y Zootecnia, Campus Ciencias Agropecuarias, Universidad Autónoma de Nuevo León, Mariano Escobedo, Nuevo León C.P. 66054, Mexico; alejandra.pescadorg@uanl.edu.mx (A.M.P.-G.); juan.zaraterm@uanl.edu.mx (J.J.Z.-R.); pablo.villarrealvl@uanl.edu.mx (J.P.V.-V.); uziel.castillovl@uanl.edu.mx (U.C.-V.); ruben.cervantesvg@uanl.edu.mx (R.C.-V.); 2 Laboratorio de Patología Molecular y Experimental, Facultad de Ciencias Biológicas, Universidad Autónoma de Nuevo León, Ave. Universidad, S/N, Ciudad Universitaria, San Nicolas de los Garza, Nuevo León C.P. 66455, Mexico; lucio.galavizsl@uanl.edu.mx; 3 Facultad de Agronomía, Campus Ciencias Agropecuarias, Universidad Autónoma de Nuevo León, Mariano Escobedo, Nuevo León C.P. 66054, Mexico; sergio.bernalgr@uanl.edu.mx

**Keywords:** dairy farming, ruminant, *Leptospira* spp., *L. interrogans*, *L. borgpetersenii*, zoonosis, veterinary public health, livestock biosecurity, seroprevalence, MAT

## Abstract

Leptospirosis is a globally significant zoonosis affecting animal health, productivity, and the environment. While typically associated with tropical climates, its persistence in semi-arid regions such as La Laguna, Mexico—characterized by low humidity, high temperatures, and limited water sources—remains poorly understood. Although these adverse environmental conditions theoretically limit the survival of *Leptospira*, high livestock density and synanthropic reservoirs (e.g., rodents) may compensate, facilitating transmission. In this cross-sectional study, blood sera from 445 dairy cows (28 herds: 12 intensive [MI], 16 semi-intensive [MSI] systems) were analyzed via microscopic agglutination testing (MAT) against 10 pathogenic serovars. Urine samples were cultured for active *Leptospira* detection. Risk factors were assessed through epidemiological surveys and multivariable analysis. This study revealed an overall apparent seroprevalence of 27.0% (95% CI: 22.8–31.1), with significantly higher rates in MSI (54.1%) versus MI (12.2%) herds (*p* < 0.001) and an estimated true seroprevalence of 56.3% (95% CI: 50.2–62.1) in MSI and 13.1% (95% CI: 8.5–18.7) in MI herds (*p* < 0.001). The Sejroe serogroup was isolated from urine in both systems, confirming active circulation. In MI herds, rodent presence (OR: 3.6; 95% CI: 1.6–7.9) was identified as a risk factor for *Leptospira* seropositivity, while first-trimester abortions (OR:10.1; 95% CI: 4.2–24.2) were significantly associated with infection. In MSI herds, risk factors associated with *Leptospira* seropositivity included co-occurrence with hens (OR: 2.8; 95% CI: 1.5–5.3) and natural breeding (OR: 2.0; 95% CI: 1.1–3.9), whereas mastitis/agalactiae (OR: 2.8; 95% CI: 1.5–5.2) represented a clinical outcome associated with seropositivity. Despite semi-arid conditions, *Leptospira* maintains transmission in La Laguna, particularly in semi-intensive systems. The coexistence of adapted (Sejroe) and incidental serogroups underscores the need for targeted interventions, such as rodent control in MI systems and poultry management in MSI systems, to mitigate both zoonotic and economic impacts.

## 1. Introduction

Leptospirosis is a neglected zoonotic disease of global importance caused by spirochetes of the genus *Leptospira* (family *Leptospiraceae*) [1]. This pathogen exhibits remarkable genomic diversity, determining variations in its pathogenicity, antigenicity, host adaptation, and environmental survival [2,3]. Phylogenetically, it is divided into three clades: pathogenic (e.g., *L. interrogans*, *L. borgpetersenii*), responsible for disease in animals and humans; intermediate (e.g., *L. santarosai*, *L. fainei*), with mild or uncertain pathogenicity; and saprophytic (e.g., *L. biflexa*), non-pathogenic species [4,5].

Regarding the serological classification of these organisms, pathogenic species are grouped into more than 300 serovars, which are then organized into 24 serogroups based on the presence of outer membrane lipopolysaccharide antigens [6,7].

Epidemiological categorization has revealed two distinct classifications of these serogroups: host-adapted serogroups, associated with chronic infections in natural reservoirs, and incidental serogroups, which manifest acute disease in non-reservoir hosts [8,9]. In cattle, host-adapted serogroups (e.g., Sejroe) have been shown to evade the immune response [10], affecting the reproductive and renal tracts. This results in prolonged dissemination through urinary or genital excretions [11,12]. Clinical manifestations include reproductive disorders (abortions, stillbirths, infertility), mastitis, agalactia, and reduced milk production. Small dairy farms suffer economic losses due to these conditions, reaching up to USD 2000 per cow per year [13,14,15,16,17].

In Mexico and Latin America, the disease reduces productivity by 20–30%, disproportionately impacting small-scale systems with limited resources [18,19,20]. Bovine leptospirosis is prevalent in Mexico [21,22,23]. Still, studies integrating bacterial isolation and risk factor analysis are scarce, particularly in semi-arid regions where environmental conditions (low humidity, high UV radiation) theoretically limit transmission yet paradoxically coexist with high livestock density [24,25]. The survival of *Leptospira* is subject to climatic variations. Tropical climates (high humidity, temperatures) favor bacterial proliferation, while arid regions challenge persistence due to desiccation and UV exposure. However, the pathogen can persist in animal reservoirs or moist microhabitats [24,25], suggesting that ecological and management factors may override climatic constraints in semi-arid zones.

The “Comarca Lagunera”—a key dairy-producing region in Mexico—exemplifies this paradox. Despite its semi-arid climate, factors such as intensive management, lack of biosecurity, and synanthropic fauna (e.g., rodents) may facilitate *Leptospira* circulation [26,27]. This study is the first in La Laguna to concurrently evaluate seroprevalence, bacterial isolation, and risk factors in dairy cattle, addressing critical gaps in understanding the epidemiology of leptospirosis in semi-arid agroecosystems. This study aimed to (1) assess seroprevalence and identify circulating *Leptospira* serogroups in intensive and semi-intensive dairy production systems, (2) evaluate system-specific risk factors, and (3) isolate viable strains to confirm environmental transmission. The results are expected to guide targeted control measures in comparable regions where climatic conditions may mask zoonotic transmission risks.

## 2. Materials and Methods

### 2.1. Study Area

The study was conducted in dairy herds from La Laguna region, spanning Durango and Coahuila states in northeastern Mexico (coordinates: 24°12′48.6″ N, 103°10′58.1″ W). The area has a semi-arid climate (mean annual precipitation: 500 mm; relative humidity: 43%) and covers 47,888 km^2^ (2.4% of Mexico’s surface area) across 15 municipalities. La Laguna is the largest dairy cattle inventory in northern Mexico (~547,240 heads; 20.2% of the national herd), with a daily milk production of ~9 million liters [28,29].

### 2.2. Study Design, Population, and Sample Collection Strategy

A cross-sectional study was conducted in dairy herds representative of the predominant management systems in La Laguna, including semi-intensive (*n* = 16) and intensive (*n* = 12) production systems. Herd selection was performed through convenience sampling (based on owners’ voluntary participation) between 2022 and 2023. To ensure representativeness, all selected herds were registered in Mexico’s national brucellosis (NOM-041-ZOO-1995) and tuberculosis (NOM-031-ZOO-1995) eradication campaigns, supervised by the Agrifood Health, Safety, and Quality National Service (SENASICA), and had remained free of both diseases for ≥1 year before sampling. The minimum sample size was estimated at 384 animals (expected prevalence: 50%, 95% CI, 5% error) [30]. We collected 445 samples (semi-intensive: 157; intensive: 288) from 28 herds to account for potential clustering effects. Within each herd, lactating cows were randomly selected during milking. Depending on the size of the lactating population, animals were sampled systematically at intervals of either every 3 or every 5 cows until the pre-established sample size per herd was reached.

Due to the known impact of vaccination on serological responses to *Leptospira* serogroups, comprehensive vaccination records were obtained for each animal and herd:

- Intensive herds received routine vaccinations against IBR, BPIV-3, BRSV, BVD, and five *Leptospira* serogroups (Canicola, Icterohaemorrhagiae, Grippotyphosa, Sejroe serovar Hardjoprajitno, and Pomona) (Leptoferm^®^ 5, Zoetis, Parsipanny-Troy Hills, NJ, USA). No leptospirosis vaccinations had been administered during the 6 months prior to sampling.
- Semi-intensive herds were unvaccinated against leptospirosis, except for a single herd that had received Bayovac^TM^ Lepto HB (Sejroe serogroup serovar HardjoBovis, Bayer, Leverkusen, Germany), with the last dose administered 4 months before sample collection.

### 2.3. Serological Diagnosis

Blood samples were collected via coccygeal venipuncture (3–5 mL) in anticoagulant-free tubes, with the serum separated, aliquoted, and stored at −20 °C until analysis. Antibodies against *Leptospira* were detected using a microagglutination test (MAT) using live reference antigens (8 serogroups comprising 10 serovars; Table 1) [31], maintained in EMJH medium (Difco^TM^, BD Company, East Rutherford, NJ, USA) at 28 °C.

MAT was performed according to a standardized protocol: initial screening at 1:100 dilution (considering ≥ 50% agglutination as reactive), followed by endpoint titration (two-fold serial dilutions from 1:200 to 1:800) of reactive samples. Seropositivity was determined using strict criteria: (a) for unvaccinated animals, titers ≥1:100 were considered positive (reported sensitivity: 80%; specificity: 90% at this cut-off [3,32,33]); (b) in vaccinated animals, positivity required either titers ≥1:800 (reported sensitivity: 60%; specificity: 95% [34]) or reactivity to non-vaccine serogroups [3,32,33,34]; and (c) coinfection was defined when sera reacted to ≥2 serogroups with the same titration [35].

To minimize bias, laboratory personnel were blinded to all epidemiological data, including vaccination status, during MAT and interpretation.

### 2.4. Bacteriological Diagnosis

Urine samples were obtained after intramuscular administration of a diuretic at 0.5 mg/kg (DIURIDE 500^@^, Agrovet Market, Lim, Peru), protected from light, and cultured in EMJH medium supplemented with nalidixic acid (Sigma-Aldrich^®^, St. Louis, MO, USA) (1:10) [32]. Cultures were incubated at 30 °C for 6 months, with weekly monitoring using darkfield microscopy (10×). Motile spirochetes were confirmed as *Leptospira* through MAT serotyping [35].

### 2.5. Epidemiological Survey and Statistical Analysis

To identify potential risk factors associated with *Leptospira* seropositivity, an epidemiological survey was conducted using a structured questionnaire (32 questions) administered in person by trained interviewers (AMP-G and JFC-S) to minimize bias. The questionnaire evaluated the following:

- Herd management: Vaccination history (frequency, types), breeding practices (natural/artificial), biosecurity measures (e.g., quarantine for new animals, vector control), and cohabitation with domestic/wild animals.
- Environmental factors: Water sources (origin, treatment), animal density (corral dimensions, animals per pen), and contact with wildlife (rodents, poultry) or domestic species (dogs, pigs).
- Reproductive health: Abortion history (frequency, trimester, disposal of fetuses/placentas), and records of mastitis/agalactiae or other reproductive disorders (retained placenta, infertility).

First, apparent seroprevalence was estimated based on the proportion of seropositive animals relative to the total number of sampled animals within each management system, with a 95% confidence interval. To account for the imperfect sensitivity and specificity of MAT, the true prevalence was estimated using the Rogan–Gladen correction estimator [36].

$$P_{ture}=\frac{P_{apparent}+Sp-1}{Se+Sp-1}$$

where *P*_apparent_ is the observed seroprevalence. The parameters for Se and Sp were stratified by vaccination status:

Unvaccinated animals (cut-off ≥ 1:100): Se = 80%, Sp = 90% [3];
Vaccinated animals (cut-off ≥ 1:800): Se = 60%, Sp = 95% [33].

Estimates were calculated separately for intensive (MI) and semi-intensive (MSI) herds to reflect system-specific differences in vaccination coverage. Confidence intervals for true prevalence were derived using the delta method [37]. During risk factor analysis, variables were separated into two categories—(1) exposure variables (contact with domestic and wild animals, herd managements, veterinary assistance, sanitary control) and (2) infection-associated outcomes (reproductive problems and clinical outcomes)—which were analyzed using separate models. For both categories, the risk factor analysis was conducted in two stages. (1) Univariable analysis was conducted using a chi-square test, where variables that demonstrate a *p*-value < 0.2 were selected for a second stage analysis. For exposure variables, a backward stepwise logistic regression multivariable analysis with 95% significance was conducted [38], while for infection-associated outcomes, an odds ratio analysis was performed. Univariable and multivariable analyses were implemented separately for both management systems (semi-intensive and intensive systems). All statistical analyses were conducted using the SPSS software v25 (IBM, Armonk, NY, USA).

### 2.6. Ethical and AI Disclosure

The study was approved by the Bioethics and Welfare Committee of the Faculty of Veterinary Medicine, Autonomous University of Nuevo León (Protocol 059/2022). AI tools assisted with language editing and manuscript structuring. The authors verified all content for accuracy. No AI was used for data generation, analysis, or interpretation.

## 3. Results

### 3.1. Serology

Overall seroprevalence was 27.0% (120/445). At the herd level, seroprevalence varied according to production system, with 100% (16/16) in semi-intensive herds and 83.33% (10/12) in intensive herds. After adjusting for the imperfect sensitivity and specificity of MAT, the estimated true prevalence of *Leptospira* seropositivity was higher than the apparent prevalence in both production systems (Table 2). In intensive herds, the true prevalence was 13.1% (95% CI: 8.5–18.7), compared to an apparent prevalence of 12.2%. Semi-intensive herds showed a true prevalence of 56.3% (95% CI: 50.2–62.1), exceeding the apparent prevalence of 54.1%.

Antibodies against all eight *Leptospira* serogroups tested were detected, with titers ranging from 1:100 to 1:800 in both production systems. Most of the seropositive animals (104/445) had lower titers (1:100 and 1:200). Titers of 1:400 were observed in 15 animals (13 from semi-intensive and 2 from intensive systems), while 14 animals (7 from each system) showed titers of 1:800. The Pyrogenes serogroup was the most frequent in semi-intensive systems (36.5%) while, in intensive systems, the Sejroe serogroup predominated (45.7%) (Table 3).

A total of nine animals—eight from semi-intensive systems and one from an intensive system—showed coinfection with two or more *Leptospira* serogroups (Table 4). The Pyrogenes serogroup was observed in seven of these cases, including one animal coinfected with three serogroups (Pyrogenes, Sejroe, and Pomona). The Sejroe serogroup was the second-most frequent coinfection, being observed in five cases.

### 3.2. Bacteriological Diagnosis and Serotyping

Only 0.5% (2/445) of all urine samples analyzed showed morphology and characteristic movement of *Leptospira* observed through darkfield microscopy. One of the samples came from an animal from a semi-intensive production system, vaccinated three months before sampling. Isolation was obtained 17 days after culturing in liquid EMJH medium. The second sample came from an animal from an intensive production system, vaccinated six months before sampling. Isolation was achieved 90 days after culture. Both isolates reacted against Sejroe serogroup antisera.

### 3.3. Risk Factors

The univariable analysis for the variables associated (<0.2) with seropositivity to one or more *Leptospira* serogroups in semi-intensive and intensive dairy cattle are presented in Table 5.

In both management systems, the univariable analysis revealed an association between seropositivity to Leptospira and coexistence with domestic and wild animals, reproductive management, reproductive problems, and biosecurity deficiencies.

In addition, Table 6 presents risk factors identified through multivariable analysis using logistic regression. Risk factors in the semi-intensive systems included coexistence with hens (OR:2.7) and natural breeding as reproductive practice (OR:2.0). Risk factors in the intensive systems included the presence of rodents in pens (OR:3.6). Table 7 presents the association between infection disorders and seropositivity against *Leptospira* in semi-intensive and intensive management herds.

## 4. Discussion

This study provides the first evidence of *Leptospira* seroprevalence, isolation, and risk factors in dairy cattle from Mexico’s Laguna region. Considering the importance of dairy cattle farming and the ecological characteristics in this region, the results obtained provide an important overview of this neglected zoonosis and emphasize the need to reinforce biosecurity measures due to the high seroprevalence, the detected risk factors, isolation, and their possible effects on dairy production in this region of northeastern Mexico. The high true seroprevalence in semi-intensive systems (56.3% vs. 13.1% in intensive) aligns with global trends in tropical dairy farms [39,40,41]. This may reflect the following factors: (i) environmental exposure, for example, pasture-based management increases contact with contaminated water/soil, especially during raining season [42,43,44]; (ii) biosecurity gaps—for example, semi-intensive systems provide less lower control over rodents/wildlife access compared to intensive systems (Table 6); (iii) regional ecology, for example, soil characteristics, microenvironments, or other unknown factors in La Laguna may prolong *Leptospira* survival outside of hosts [40,41]. Notably, our findings diverge from those found in semi-arid regions like Tanzania, where lower seroprevalence may reflect environmental constraints on bacterial survival [39]. However, shared risks, such as rodent infestations and water-source contamination, highlight universal control challenges [39,45]. While rodents were reported in both systems, their contact rates may differ; intensive systems’ concrete floors could limit bacterial persistence compared to earthen pastures [43].

Notably, the detection of diverse serologic coinfections in our study (e.g., Pyrogenes + Sejroe, Pyrogenes + Tarassovi) underscores the epidemiological complexity of leptospirosis in La Laguna, suggesting simultaneous exposure to multiple serological variants of *Leptospira*. This pattern likely results from overlapping reservoir habitats (rodents, dogs, wildlife) and persistent environmental contamination, creating ideal conditions for polyvalent transmission. Such coinfections amplify zoonotic risks, particularly from non-adapted serogroups such as Pyrogenes [46], and may exacerbate clinical outcomes (e.g., abortions, mastitis) [47,48]. Such multi-serogroup infections align with the epidemiological profile observed in Mediterranean dairy systems [41], where management factors (natural breeding, high animal density) and ecological drivers (rodent proliferation, water contamination) interact to sustain complex Leptospira transmission cycles. On the other hand, the predominance of non-vaccine serogroups (e.g., Pyrogenes) and the high true seroprevalence in semi-intensive herds (56.3%), despite partial vaccination coverage (6.25% of herds), suggests that current protocols may inadequately protect against circulating strains. This gap underscores the need for herd-specific strategies, including serogroup surveillance to inform tailored vaccine formulations or adjusted administration schedules. Similar challenges have been reported in dairy systems with comparable management conditions, such as smallholder farms in Brazil [10,48] and India [49], where vaccines reduced clinical signs but failed to prevent infection by non-target serogroups. The persistence of these seroreactivities suggests either incomplete cross-protection within serogroups (e.g., between Hardjo-bovis and Hardjo-prajitno), or a complete lack of protection across serogroups (e.g., Pyrogenes vs. vaccine strains), where mismatches between vaccine strains and field variants reduced control efficacy. In La Laguna, this mismatch is exacerbated by the coexistence of multiple serogroups, highlighting the importance of integrating vaccination with complementary measures (e.g., reservoir control, water sanitation) to mitigate transmission risks.

The identified risk factors reflect distinct epidemiological pressures in each production system. In intensive systems, the strong association between rodent presence (OR: 3.6) and seropositivity aligns with global reports of rodents as key reservoirs for *Leptospira* in high-density dairy operations [41,50,51]. Rodents contaminate feed, water, and bedding with urine containing viable pathogens, creating persistent transmission hotspots—a phenomenon documented in similarly managed herds in New Zealand [52] and the Netherlands [53].

The even higher risk linked to first-trimester abortions (OR: 10.1) underscores the reproductive impact of leptospirosis. This result is consistent with findings from intensive Brazilian dairy herds, where *L. interrogans* serovar Hardjo (Sejroe serogroup) caused abortion storms following fetal infection via placental colonization [47,54]. The consistency of this association across studies suggests its utility as a sentinel indicator for leptospirosis outbreaks in confined cattle populations.

For semi-intensive systems, the prominence of hens (OR: 2.8) as a risk factor may reflect their role as mechanical carriers of *Leptospira*-contaminated soil or water into cattle environments—a mechanism proposed in recent studies from Indonesia [55] and Trinidad [56]. Unlike rodents, hens are unlikely maintenance hosts but may facilitate pathogen spread through movement patterns.

In this study, clinical damage due to mastitis/agalactia (OR: 2.8) was detected as another significant association, possibly associated with the colonization of mammary tissue by pathogenic *Leptospira*, as has been demonstrated in experimental infections in goats [57]. This aligns with Chilean dairy farms, where leptospirosis-positive herds had 3.2× higher odds of subclinical mastitis [58], suggesting milk quality implications beyond reproductive losses.

Finally, natural breeding (OR: 2.0) likely increases exposure through venereal transmission or contaminated genital secretions [11,12]—a risk amplified by the lack of biosecurity in semi-intensive systems. Similar patterns were observed in Argentine beef herds, where natural mating increased seropositivity odds by 2.5× compared to artificial insemination [59].

This study has several limitations. First, the use of convenience sampling may introduce selection bias. Second, the bacterial isolation rate was low (2/445), consistent with Leptospira’s fastidious growth requirements. Our true prevalence estimates assume uniform Se/Sp across herds. However, MAT performance may vary by serogroup [34]. Future studies should incorporate serogroup-specific validation. As a cross-sectional study, temporality constraints prevent the determination of whether the associated factors were before or after the outcome (infection or disease). Additionally, vaccination-induced antibody titers can be low or transient in some animals, declining within 3–6 months post-vaccination; therefore, the microscopic agglutination test (MAT) may fail to detect vaccinated animals if sampling occurs outside this period. Interactions between risk factors were not tested due to sample size constraints, which should be explored in future studies. Future research should also incorporate molecular methods (e.g., PCR and whole-genome sequencing) to improve isolate characterization. Despite these limitations, our findings support One Health strategies, including rodent control, expanded vaccination programs, and producer education.

## 5. Conclusions

This study demonstrated that semi-intensive dairy cattle systems exhibit a 4-fold higher true seroprevalence of leptospirosis than intensive systems (56.3% vs. 13.1%), with risk factors including rodent exposure, poultry cohabitation, and natural breeding practices. The isolation of Sejroe serogroup confirms active *Leptospira* circulation despite semi-arid conditions. System-specific risk patterns for *Leptospira* seropositivity were identified: in intensive systems, seropositive animals were strongly associated with rodent infestation (OR: 3.6) and first-trimester abortions (OR: 10.1) while, in semi-intensive systems, seropositivity was predominantly linked to poultry contact (OR: 2.8), mastitis occurrence (OR: 2.8), and natural breeding (OR: 2.0). These findings promote the following approaches: (1) tailored vaccination programs targeting predominant serogroups (Sejroe/Pyrogenes), (2) enhanced rodent control measures in confined operations, and (3) farmer education on zoonotic transmission risks. The results underscore the need for differentiated control strategies to reduce both animal infection and public health risks in northern Mexico’s dairy basin, with potential applications to similar production systems globally.

## Figures and Tables

**Table 1:** *Leptospira* strains used as antigens during the MAT test.

Species	Serogroup	Serovar	Strain
*L. interrogans*	Australis	Brastislava	Jez-Bratislava
	Canicola	Canicola *	Hond Utech IV
	Icterohaemorrhagiae	Icterohaemorrhagiae *	RGA
	Pomona	Pomona *	Pomona
	Pyrogenes	Pyrogenes	Salinem
	Sejroe	Hardjo *	Hardjo-prajitno
	Sejroe	Wolffi	3707
*L. borgpetersenii*	Tarassovi	Tarassovi	Pepereletsin
	Sejroe	Hardjo *	Hardjo-bovis
*L. kirschneri*	Grippotyphosa	Grippotyphosa	Moskva V

* Serovars included in the vaccine.

**Table 2:** True versus apparent seroprevalence of *Leptospira* in dairy cattle from La Laguna, Mexico: significant differences between herd management systems.

Herd System	Sampled	Seropositive	Apparent Prevalence	95% CI	True Prevalence	95% CI
Semi-intensive	157	85	54.1%	46.4–61.9	56.3%	50.2–62.1
Intensive	288	35	12.2%	8.4–15.9	13.1%	8.5–18.7
Total	445	120	27.0%	22.8–31.1		

**Table 3:** Distribution of *Leptospira* serogroups among 85 and 35 seropositive dairy cattle in semi-intensive and intensive herds, respectively, in La Laguna, Mexico.

Serogroup	Semi-Intensive (%)	Intensive (%)	Total (%)
Pyrogenes	31 (36.5)	11 (31.4)	42 (35.0)
Sejroe	30 (35.3)	16 (45.7)	46 (38.3)
Tarassovi	14 (16.5)	4 (11.4)	18 (15.0)
Grippotyphosa	2 (2.4)	1 (2.9)	3 (2.5)
Pomona	3 (3.5)	0	3 (2.5)
Canicola	6 (7.1)	0	6 (5.0)
Icterohaemorrhagiae	3 (3.5)	1 (2.9)	4 (3.3)
Australis	6 (7.1)	5 (14.3)	11 (9.2)

**Table 4:** Diverse serologic coinfections of pathogenic *Leptospira* in Mexican dairy cattle: semi-intensive herds as high-risk hotspots.

Serogroup Combination	Seroreactives		Coinfections
	Semi-Intensive	Intensive	
Pyrogenes + Sejroe	3	0	3
Pyrogenes + Tarassovi	1	1	2
Pyrogenes + Canicola	1	0	1
Sejroe + Tarassovi	1	0	1
Sejroe + Canicola	1	0	1
Pyrogenes + Sejroe + Pomona	1	0	1
Total	8	1	9

**Table 5:** *Leptospira* seropositivity risk factors in dairy cattle: divergent clinical and environmental associations in semi-intensive vs. intensive herds (La Laguna, Mexico).

Variable	Serological Status		*X* ^2^	*p*-Value	OR (95% CI)
	Positive (%)	Negative (%)			
Semi-intensive					
Coexistence with hens			8.9	0.003	2.8 (1.5–5.3)
Yes	51 (32.5)	26 (16.6)			
No	34 (21.7)	46 (29.2)			
Mastitis/agalactiae			9.63	0.002	2.8 (1.5–5.2)
Yes	53 (33.8)	27 (17.2)			
No	32 (20.4)	45 (28.6)			
Natural breeding			4.42	0.03	2.0 (1.1–3.9)
Yes	62 (39.5)	41 (26.1)			
No	23 (14.6)	31 (19.7)			
Coexistence with dogs			1.83	0.18	0.6 (0.3–1.2)
Yes	54 (34.4)	53 (33.8)			
No	31 (19.7)	19 (12.1)			
Coexistence with wild pigs			2.33	0.13	1.6 (0.9–3.0)
Yes	47 (29.9)	31 (19.7)			
No	38 (24.2)	41 (26.1)			
Coexistence with cats			2.49	0.11	0.6 (0.3–1.1)
Yes	33 (21.0)	37 (23.6)			
No	52 (33.1)	35 (22.3)			
Coexistence with domestic animals			2.93	0.09	1.9 (0.9–3.8)
Yes	49 (31.2)	51 (32.5)			
No	36 (22.9)	21 (13.4)			
>100 animals per herd			2.26	0.13	0.6 (0.4–1.2)
Yes	37 (23.56)	40 (25.5)			
No	48 (30.6)	32 (20.4)			
Coexistence with domestic pigs			1.83	0.18	0.6 (0.3–1.2)
Yes	44 (28.0)	45 (28.7)			
No	41 (26.1)	27 (17.2)			
Intensive					
Abortions in first-trimester pregnancy			36.04	<0.001	10.1 (4.2–24.2)
Yes	28 (9.7)	72 (25.0)			
No	7 (2.4)	181 (62.9)			
Presence of rodents			10.8	0.001	3.6 (1.6–7.9)
Yes	26 (9.0)	113 (39.2)			
No	9 (3.1)	140 (48.6)			
Vector control			3.08	0.08	0.5 (0.3–1.1)
Yes	13 (4.5)	134 (46.5)			
No	22 (7.6)	119 (41.3)			
*Leptospira* vaccination			1.80	0.18	0.6 (0.3–1.2)
Yes	17 (5.9)	153 (53.1)			
No	18 (6.2)	100 (34.7)			
Annual veterinary assistance			1.97	0.16	0.6 (0.3–1.2)
Yes	13 (4.5)	126 (43.8)			
No	22 (7.6)	127 (44.1)			
Abortion in second trimester pregnancy			2.17	0.08	2.1 (0.8–5.6)
Yes	5 (1.7)	65 (22.6)			
No	30 (10.4)	188 (65.3)			
Abortion in third trimester pregnancy			1.75	0.19	1.6 (0.6–4.0)
Yes	6 (2.1)	70 (24.3)			
No	29 (10.1)	183 (63.5)			
Liming placenta			2.90	0.09	0.5 (0.3–1.1)
Yes	15 (5.2)	147 (51.0)			
No	20 (6.9)	106 (36.8)			
Placental burying/burning			3.49	0.06	0.4 (0.2–1.0)
Yes	7 (2.4)	91 (31.6)			
No	28 (9.7)	162 (56.3)			
Placental disposal			3.38	0.07	0.4 (0.2–1.1)
Yes	6 (2.1)	82 (28.5)			
No	29 (10.1)	171 (59.4)			
Artificial Insemination			2.33	0.13	0.6 (0.3–1.2)
Yes	11 (3.8)	114 (39.6)			
No	24 (8.3)	139 (48.3)			

**Table 6:** Risk factors associated with leptospirosis seropositivity among 85 and 35 seropositive dairy cattle in semi-intensive and intensive herds, respectively, in La Laguna, Mexico.

Management System	Variable	OR	*p*-Value	95% CI
Semi-intensive				
Coexistence with hens		2.8	<0.001	1.5–5.3
Natural breeding		2.0	<0.001	1.1–3.9
Intensive				
Presence of rodents		3.6	0.003	1.6–7.9

**Table 7:** Infection disorders associated with leptospirosis among 85 and 35 seropositive dairy cattle in semi-intensive and intensive herds, respectively, in La Laguna, Mexico.

Clinical Disorder	OR	*p*-Value	95% CI
Semi-intensive			
Mastitis/agalactiae	2.8	0.002	1.5–5.2
Intensive			
Abortion during first trimester of pregnancy	10.1	<0.001	4.2–24.2

OR: odds ratio; CI: confidence interval.

## Data Availability

The original contributions presented in this study are included in the article. Further inquiries can be directed to the corresponding authors R.A.-R. or J.F.C.-S.
